# Children’s, parents’, and teachers’ experiences of the feasibility of a telerehabilitation intervention for children with acquired brain injury in the chronic phase – a qualitative study of acceptability and participation in the Child In Context Intervention (CICI)

**DOI:** 10.1186/s12913-023-09589-z

**Published:** 2023-06-08

**Authors:** Edel Jannecke Svendsen, Eli Marie Killi, Nina Rohrer-Baumgartner, Ingvil Laberg Holthe, Maria Sandhaug, Ida M. H. Borgen, Shari L. Wade, Solveig Laegreid Hauger, Marianne Løvstad, Line Kildal Bragstad

**Affiliations:** 1 Department of Research, Sunnaas Rehabilitation Hospital, Nesodden, Norway; 2 Oslo Metropolitan University, Oslo, Norway; 3 CHARM - Research Centre for Habilitation and Rehabilitation Models & Services, Institute of Health and Society, Faculty of Medicine, University of Oslo, Oslo, Norway; 4 Department of Profession Strategy and Knowledge Brokering, Norwegian Service for Special Needs Education, Oslo, Norway; 5 Department of Psychology, Faculty of Social Sciences, University of Oslo, Oslo, Norway; 6 Department of Physical Medicine and Rehabilitation, Oslo University Hospital, Oslo, Norway; 7 Division of Physical Medicine and Rehabilitation, Cincinnati Children’s Hospital Medical Center, 3333 Burnet Ave, OH 45229 Cincinnati, USA; 8 University of Cincinnati College of Medicine, OH Cincinnati, USA

**Keywords:** Feasibility, Children, Rehabilitation, Acquired brain injury, Qualitative research, School intervention, Family-centered, Tele-rehabilitation

## Abstract

**Background:**

This is a qualitative feasibility study of the Child in Context Intervention (CICI). The CICI is an individualized, goal-oriented and home-based tele-rehabilitation intervention which targets everyday functioning of children (6–16 years) with acquired brain injury in the chronic stage, and their families, one year or more after insult, who have ongoing challenges (physical, cognitive, behavioral, social and/or psychological). The aim of this study is to better understand how children, parents and teachers experienced participation and acceptability; to develop knowledge about the mechanisms of change, and to explore how the CICI was tailored to the context.

**Methods:**

Six families and schools participated in the intervention, which comprised seven tele-rehabilitation sessions in which the child and parent participated, one in-person parent seminar and four digital school meetings. A multidisciplinary team delivered the intervention to 23 participants over a 4- to 5-month period. The intervention involved psychoeducation about targeted acquired brain injury-related problems, such as fatigue, pain, or social challenges. All but one consented to participate in the current digital interview study. The data were analyzed using content analysis.

**Results:**

The experience of participation and acceptability varied among the children. Attendance was consistently high; the child participants felt mostly listened to and could influence goal setting and strategies. However, engaging and motivating the child participants proved somewhat challenging. The parents found the CICI rewarding, useful and relevant. However, they had different experiences regarding which intervention component they perceived as most helpful. Some argued in favor of the ‘whole intervention’, while others highlighted new knowledge, SMART goals or the school collaboration. The teachers found the intervention acceptable and useful but wanted a better meeting plan. They had difficulties in finding time for meetings, emphasized the involvement of school leaders, and appreciated the digital format.

**Conclusions:**

Overall, the intervention was perceived as acceptable, and the participants felt that the various intervention components contributed to improvements. The CICI’s flexibility facilitated tailoring to different contexts based on the children’s functional level. The digital format saved time and provided flexibility regarding the amount of attendance but limited full participation from children with more severe cognitive impairments.

**Trial registration:**

ClinicalTrials.gov Identifier: NCT04186182.

## Background

Schoolchildren with acquired brain injury (ABI) may suffer long-lasting physical, cognitive, behavioral, and social symptoms, creating a wide range of rehabilitation challenges that require an individualized, context-sensitive and interdisciplinary approach [1]. Studies have also indicated that younger children with ABI experience difficulties in developing academic skills, accompanied by a lack of effective school support [2, 3]. Rehabilitation for this group should involve a range of activities— including educational and psychosocial support, delivered by a multidisciplinary expert team in a person-centered process [4, 5]. Only a few rehabilitation trials tailored for schoolchildren with ABI in the chronic phase and their families are available, and a lack of convincing treatment recommendations remains a challenge in the field. There is convincing evidence for caregiver-focused interventions, but complex ABI-rehabilitation interventions where children, parents and schools all participate have not been evaluated in trials [6].

The involvement of children in clinical planning and decision-making has been lacking [7], even though supporting children to actively participate in the treatment may improve outcomes, create more personalized care and contribute to better management of their conditions [8, 9]. To facilitate children’s engagement in the rehabilitation process, the aims and contents must be perceived as meaningful to the child. Herein, the child’s preferences need to be taken into account [10]. The extent to which the child’s involvement is possible will depend on their development stage, type and severity of illness, personal characteristics and patient relationships with professionals [8, 9]. In addition, children with ABI are at greater risk of missing out on participation in different arenas as compared to their non-injured equals [11]. A recent review showed that obstacles to child participation in brain-related healthcare are related to children’s level of understanding, the time and energy necessary for information processing and the lack of perceived relevance of the information. Participation in treatment has two components: *attendance*, defined as ‘being there’ and measured as the frequency of attending, and *involvement*, the experience of participation while attending [12]. Treatment participation for children is supported by parents, which highlights the importance of family involvement in pediatric rehabilitation. Given the heterogeneity in age, awareness of deficits, injury severity and types of impairment for children with ABI, their ability to be involved varies. Nevertheless, even young children with severe injuries should have the right to participate [13].

Qualitative research can help explore patient perspectives prior to a clinical trial to ensure that the intervention is acceptable and experienced as relevant to the needs of the patients and their families [14]. Despite being overlooked in the past, the value of feasibility testing is now widely accepted [15]. For example, it is helpful to understand whether a specific intervention can be conducted in an acceptable manner [16]. Acceptability is a multi-faceted construct that reflects the extent to which people receiving an intervention consider it appropriate, based on their experienced cognitive and emotional responses to it [17]. Participant responsiveness is also related to the intervention’s mechanisms of change (i.e. how the delivered intervention may be experienced to result in positive change), as experienced by the participants [18, 19]. What interventionists perceive to be the expected mechanism of change may be different from what individual families perceive as helpful. This may be especially true for families of children with ABI, where the concerns are diverse and heterogeneous, further supporting the use of qualitative data. The understanding of participants’ experiences about what aspect of an intervention they experienced as contributing to improvement can enhance the design of the intervention and help researchers better understand whether the mechanisms of change are reflected in the perceived benefits. Hence, it can benefit future evaluations and improve patient-centered healthcare [20], as well as enable researchers to optimize the intervention outcome or conduct of the trial [21].

### The Child in Context Intervention

The Child in Context Intervention (CICI) was developed to enhance the participation and everyday functioning of children with ABI in the chronic stage in the home and school environments, as well as to improve parent functioning. The CICI was modelled after two studies conducted for adults with traumatic brain injury [22, 23] and adapted for children, families, and schools. For a full description of the study protocol, please refer to Rohrer-Baumgartner et al.’s paper [24]. The intervention targets problem areas that a specific child and their family experience as most challenging, with the aim of providing the families with strategies that would benefit them even after the trial completion [25]. It was designed in line with current recommendations in child ABI research and the Medical Research Council’s framework for evaluating complex interventions [15, 26, 27].

The intervention components included seven tele-rehabilitation sessions conducted using videoconferencing between CICI therapists and the family, one parent seminar and four digital school meetings, which were attended by the child’s key personnel at school. Parents were also encouraged to attend school meetings. Children were allowed to participate in the school meetings if they wished to, but none chose to attend. The families and schools received a handbook containing different topics about usual challenges for families following ABI (CICI handbook). The components were designed to facilitate an increased understanding of symptoms through written and oral psychoeducation (handbook and interactive lectures about child symptoms) to help set goals of relevance to everyday functioning and to create a shared understanding of the child’s needs and improved cooperation between schools and families.

The intervention was delivered by a multidisciplinary team comprising one special needs teacher, one specialized pediatric nurse (E.J.S.) and two clinical neuropsychologists (N.R.-B. and I.L.H.) with clinical experience in family-centered care and rehabilitation. The therapists ensured child involvement and building alliances with the children. Meetings between the therapists and participants were conducted digitally to reduce the burden of travel, time spent and costs, actualized by Covid-19. The feasibility of the outcome measures, neuropsychological measures and quantitative feasibility data can be found in the paper by Laberg Holthe et al. [28].

At baseline, the children and parents nominated the three problems in their everyday life related to ABI and rated how difficult they perceived them to be. Goals were developed following the SMART principle, ensuring that they were Specific, Measurable, Attainable, Realistic and Timely (SMART) [29]. During the family sessions, 3–5 SMART goals were developed, goal attainment scaling was performed [30] and strategies were developed. School-relevant goals were presented to the children’s teaching personnel, where school-based strategies were negotiated, implemented, and evaluated during school meetings.

Table 1 presents an overview of the hypothesized mechanisms of change and desired outcomes. It was hypothesized that the combination of treatment components would result in positive change for the families, and that one mechanism of change would be the establishment of individualized SMART goals and treatment strategies in the child’s context. The parent seminar was designed to facilitate the sharing of experiences, practical advice and emotional support. The one-day seminar was expected to result in enhanced motivation to engage in the intervention and parenting self-efficacy.

**Table 1:** Overview of the intervention's presumed mechanisms of change and outcome

Mechanisms of change	Outcome
*Assumptions about how the delivered intervention produces positive change*	*Desired outcomes and change*
**CICI-specific mechanisms**: • Individualized and family-centered SMART goals based on the main ABI-related problems for families • Developing, adjusting, and implementing treatment strategies in the child’s context (at home and in school) • Increasing knowledge/understanding of symptoms through written and oral psychoeducation (CICI handbook/material and interactive lectures about common child ABI symptoms) • Promoting a shared understanding of children’s needs and improved collaboration between schools and families • Sharing experiences and feelings with other parents in a comparable situation in a therapist-led environment to enhance intervention motivation and parenting self-efficacy **General mechanisms**: • Degree of motivation, participation and involvement • Participants’ experiences of alliance and support • Therapists’ competencies and background • Participants’ resources and backgrounds • School resources and facilities	• Enhanced participation and everyday functioning of the child in everyday life and school • Improved family functioning • Increased child self-efficacy • Increased parenting self-efficacy • Decreased burden of ABI symptoms for child and parents

### Aims and objectives

The aim of this study was to better understand how children, parents and teachers experienced participation and acceptability, to develop knowledge about the mechanisms of change, and to explore how the CICI was tailored to the context. This was done by investigating the following:

1. How the participants experienced their own involvement and participation in the intervention.
2. How and whether the CICI was experienced as acceptable to children, parents, and schools, and thereby, which potential mechanisms of change were perceived as beneficial by the participants.

## Design and methods

The feasibility study comprised a one-group pre-post design, with a pre-intervention baseline assessment (T1) and a follow-up assessment after the intervention period of 4–5 months (T2) and qualitative interviews. The feasibility study was first launched in January 2020. Due to a Covid-19-related lockdown, it was put on hold, then re-opened in August 2021 and concluded in December 2021. The quantitative results are published in Holthe et al.’s paper [28]. Here we report on the qualitative part of the feasibility study.

### Participants

Six children and their parents were recruited from Sunnaas Rehabilitation Hospital. The inclusion criteria were as follows: (1) 6–15 years old; (2) CT-or MRI-verified ABI diagnosis; (3) at least one year since onset; (4) self- or parent-reported ABI-related subjective cognitive, emotional or behavioral problems influencing everyday life and/or participation related to family, friends, school or local community; (5) regular school attendance and (6) ability and willingness to participate actively in the rehabilitation intervention.

The exclusion criteria were as follows: (1) severe pre- or comorbid neurological disorders, such as severe autism or uncontrolled epilepsy; (2) children with brain tumors in active treatment or at great risk of relapse; (3) children with severe psychiatric illness or in institutionalized care; (4) children in child welfare services; (5) severe parental psychiatric illness, drug abuse or indications of a history of or risk of domestic violence and (6) non-fluent in Norwegian. These criteria are discussed in Rohrer-Baumgartner et al.’s paper [24].

The feasibility study was conducted collaboratively between the specialized healthcare system, the National Service for Special Needs Education in Norway (Statped), teachers/school personnel and relevant local rehabilitation services.

### Interviews

Six children, five mothers, six fathers and six teachers (one from each school), totaling 23 participants, consented to participate, resulting in eighteen qualitative interviews performed in January 2022, 3–5 weeks after finishing the intervention. The children’s injuries were TBI (2), anoxia (2) and brain hemorrhage (2). Overall, their range of cognitive functioning varied between normal and impaired. The parents were interviewed together—except for one father, who was interviewed alone (the mother declined). In four of the children’s interviews, one or both parents participated. The children were between 11 and 15 years old at the time of recruitment (mean 12.8), and the time post injury was 1.5 to 13 years. While some of the children struggled with severe cognitive impairment, others were mildly affected. Level of verbal abstract reasoning (Similarities subtest of the WISC-V) varied from scaled scores 1–12, while nonverbal reasoning (Matrix reasoning) varied from 3 to 9 [31]. One child had neuropsychological functioning within the normal range, one child had neuropsychological functioning within the normal range except for reduced working memory (-1.3 sd), three children had considerable neuropsychological impairment within several domains (-1- -3 sd) and one child had an overall severely impaired neuropsychological functioning with all scores in the impaired range (-1,3- -3 sd). An overview over the measures used and a brief description of each child’s results can be found in Laberg Holthe, et al. [28]. Four children were in regular schools, one child attended a private school and one attended a special educational school. Except for one family that received one night of respite care for their child per week, all children lived at home with both their parents and siblings and attended school daily.

#### Interview setting

The participants were interviewed at home through the secure digital platform Cisco Webex™ using their personal computers.

### Data collection

Digital qualitative interviews, which have been shown to provide high-quality qualitative data [32], were used for data collection to help ensure social distancing requirements during the Covid-19 pandemic. Interviews are useful when investigating personal experiences and understanding a topic [33]. The semi-structured interviews applied a thematic interview guide. The themes were expectations and experiences with participation in the intervention; usefulness, benefits and concerns related to the strategies developed in intervention and experience with the digital solution and user involvement. The second author (E.K.) performed all the interviews. She has experience interviewing children and parents. As she was not part of the intervention delivery team, the participants were able to speak freely.

### Analysis

The interviews with the children lasted about 30 min each and those with parents and teachers lasted about 60 min each. The interviews were transcribed verbatim, resulting in 236 written pages. Braun and Clarke’s (2006) thematic analysis inspired the analysis conducted in this study. It consists of six phases: (1) familiarizing yourself with your data, (2) generating codes for the relevant data in the dataset, (3) searching for themes, (4) reviewing the themes, (5) defining and naming the themes and (6) producing a report. The transcripts of each group of participants (children, parents, and teachers) were coded separately and, thereafter, rearranged deductively into themes based on the research questions. Therefore, steps 3 and 4 were, performed with the pre-defined themes, with less attention paid to defining and naming the themes. Consensus was established on the interpretations and sorting into themes. Excluding the transcription part (performed by E.K.) in step 1 and coding (performed by E.J.S.) in step 2, E.J.S., E.M.K. and L.K.B. conducted steps 3–5 in a systematic process of discussion and reflection. In the results, the letter C marks quotes from children, M from mothers, F from fathers and T from teachers. To signify which of the six child–parent–teacher constellations the quotes were collected from, we added a second letter (constellation A-F). For example, CA signifies a quote collected from a child in constellation A.

### Ethics

The Norwegian regional ethics committee of Southeast (approval number REK 2019/1283) approved the study. The children were provided with oral and written age-appropriate information and verbally assented to participate, since they could not consent because of their age. Both parents gave written consent on behalf of their children. The parents and teachers received oral and written information. Parents gave written, while teachers gave oral consent to participate and for the results to be published.

## Results

In the following sub-section, the participants and their main difficulties are described; thereafter, we present their experience of participation and acceptability.

### Participants’ ABI-related problems in daily life

The main ABI-related problems reported by parents and children, are illustrated in Table 2.

**Table 2:** Overview of identified main ABI-related problems in daily life, reported by parents and children

Main ABI-related problems reported by parents	Main ABI-related problems reported by children
• Fatigue • Social challenges and isolation • Study technique • Pain/headache • Cognitive gap with peers • Lack of independence in getting around • Parenting a child with ABI • Physical challenges, such as balance, coordination and strength • Emotion regulation • Parental exhaustion tied to challenges in getting adequate help for the child	• Fatigue • Social challenges • Study skills • Pain/headache • Losing track in conversations with peers • Getting around independently • Not able to follow activities and changes in the same tempo as peers • Sleep

The established SMART goals were based on these main ABI-related problems, as reported by the families. All but one SMART goal was achieved during the intervention period. For extensive information about the goals, goal attainment scores and children’s functional profiles please refer to Holthe et al.’ paper [28].

### Participation

In what follows, we first present the children’s, followed by the parents’ and, finally, the teachers’ experiences of participation, and the degree to which they were involved and had influence.

#### Children’s experiences of study participation

Most children reported being listened to and being able to say what they wanted. For example, one child said, ‘I always felt that I could be part of the decision’ (CD). While this was also true for child A, he still felt a bit sidelined: ‘Yes, but sometimes my answers were interpreted differently than my intentions, you know’ (CA). This child felt that the parents’ opinion regarding a discussed topic were acted upon, but not his perspective on the matter. Another child with a more severe impairment had disagreed about a strategy concerning the use of earplugs (to reduce fatigue through noise reduction), but the strategy had been decided on anyhow. Another child had difficulty in answering if she had been listened to and answered: ‘To be quite honest, those meetings were pretty boring’ (CC). When asked about what she was allowed to decide, she replied, ‘I decided that … when I don’t want to do things and stuff like that’ (CC).

The children noted that participation in meetings was voluntary, but that they sometimes participated out of ‘duty’ or were talked into it. Most children said that they understood everything the therapists said, and if not, that the therapists rephrased the exchanges to improve understanding. However, the sessions involved verbal interactions among the child, the therapist and parents that may have been perceived as lengthy, boring and not relevant, particularly for children with a significant cognitive impairment. When asked how the meetings were, one child said, ‘Talked a lot, yes, almost only talking!’ (CF). When asked what could have helped, most children did not reply anything, but one child said, ‘And talk less about the ‘threads’ in my head or talk more about fun stuff or…’ (CC).

Two of the children also mentioned timing of meetings as important. One boy appreciated how all the meetings were held outside his team’s practice schedule, while one girl expressed how annoyed she was when she had to attend a meeting when there was something important, she missed out on at school.

Nonetheless, they were happy that they could leave the meetings after a while and mostly join and leave the digital meetings as they pleased. Some children said that, although they did not want to participate in all the meetings, they had agreed to attend after all.

#### Parents’ experiences of participation

All parents found that they had a high degree of influence in the CICI. Mother E is an example of how most parents evaluated their ability to participate:

‘I feel that we have had a major influence. For instance, they made a turnaround in the middle of the study just because we wanted to. Certainly, I feel like we’ve had a lot to say in it. And when we saw that things got difficult or that we needed something, they made a quick turnaround’.

One father highlighted the opportunity of influencing with the content in the meetings: ‘I believe it has been good because we have discussed both goals and strategies and what to work on. We’ve been allowed to be a part of it’ (FC). Another father said ‘I found it exciting to be a part of it. And when we’ve mentioned something, it’s been taken care of, so it’s worked out well’ (FF). The parents said that the scheduling of meetings and the time the children were required to be present were optimized.

#### Teachers’ experiences of participation

Most teachers experienced being respected and felt that they had influence in the intervention. This is exemplified by the expressions of teachers E and F:

‘I feel that we’ve been heard when we talk about the student, about everyday life and about the challenges, so we have been heard. They have been open to our feedback, adjustable. This is what creates good cooperation, and not like top-down – we have the competence and so on. (…) I really feel that we have influenced how this worked out’. (TE)

‘I felt I have had it (influence) on everything’. (TF)

Interviewer: In what way?

‘Well, I put forward challenges from the school that I had noticed and received some suggestions on how we could work on those. And when I said that something was impossible to do, we looked at other suggestions on how things could be done and agreed on something that was possible to do together’. (TF)

Although all teachers reported that they would recommend other teachers to participate in the CICI, some experienced less influence in the scheduling and rescheduling of meetings that provided more predictability. Some wanted a more explicit plan and better management of the meetings. The teachers also had difficulties in finding time for the meetings and, therefore, emphasized involving school leaders in the intervention to help them prioritize participation in the CICI school sessions.

The overall impression was that all three informant groups could influence the decisions made in the intervention, although teachers and children to a lesser extent than parents.

### Experienced acceptability of CICI

In what follows, we first give accounts of children’s, then parents ‘and, finally, teachers’ experiences of acceptability of the CICI.

#### Children’s experiences of acceptability

The children’s responses regarding the perceived usefulness of the intervention varied greatly. While a few of them struggled to talk about what had helped, others, when reminded of their goals, expressed that they had reached their goals. For some of the children, it was unclear what the CICI targeted; however, they expressed in different ways that it felt good to get help and be acknowledged by the adults. One of the children highlighted how the intervention had helped her both at home and in school: ‘I wanted to get better at talking about things. That’s what I have worked on. And then I worked on becoming better at letting others know when I need a break’ (CD). Most children said that they liked talking to the therapists, although one child with severe cognitive impairment added that the meetings were a bit boring. A second child expressed that the meetings were ‘a bit useful and a bit stupid’ (CC).

Three of the children with memory impairments had difficulties answering if they were able to do something after the intervention that they could not do before. One of these children was interviewed alone and had trouble remembering the goals on which she had worked. She gave answers like ‘I can´t remember’ (CF) and further noted that she did not have any improvement. The two remaining children, who had one of their parents accompany them during the interview, were prompted about the goals, exemplified in the following excerpt (ME and CE):

Interviewer: Can you tell me if you worked on something in the CICI study that helped you to do things you were unable to do before the study?

Child: I want mom to tell.

Mother: Do you want me to tell?

Child: Yes.

Mother: I think you now are better at thinking about and looking at what others are doing before you do things yourself, instead of saying that you do not want to join in. Now you are first observing the others and then you try.

Child: Yes.

Mother: I think it has improved a lot.

Child: Yes.

Mother: Do you agree, or….

Child: Yes.

All children were asked about the digital format of the intervention meetings; four of them said that they would have preferred face-to-face/physical meetings, while the remaining two were unsure.

#### Parents’ experiences of acceptability

All parents found the CICI highly acceptable. The parts of the intervention considered most helpful differed among the parents. Some argued in favor of the ‘whole package,’ while others highlighted the new knowledge, the SMART goals or the school collaboration.

All parents expressed that the goal-oriented approach was relevant and beneficial; to some, it was experienced as the most valuable part. The approach was described as helpful to be prompted to select specific goals to focus on, and that the therapists expected the parents to spend the time needed to work on the goals between sessions. One father put it like this:

‘The SMART goals helped to break down the problem into manageable parts. Of course, we did see all the overarching challenges but having to start with the minor things that contribute to making it better, was important. This was challenging to do on our own (…), to understand the problem’. (FB)

Several parents felt more empowered and that they had become ‘better parents’. One parent said, ‘You feel like 100 kg has been lifted from your shoulders, because you felt like you had failed as a parent, why isn’t she sleeping? Why is she still fatigued? I feel I can understand her better and help her in a better way’ (MD). New knowledge about the consequences of brain injury was especially important to parents with children in the shortest time since injury. The CICI handbook supported their understanding of the child and gave reflections and advice. Mother A put it like this: ‘We now have a clearer understanding about the brain injury and are more capable of delineating between the problems connected to sequela after the ABI, or problems separate from that’.

However, the parents of children with the longest time since injury did not think they learned anything new; rather, they were convinced that it was beneficial to receive help to stay committed to a strategy and to translate their knowledge into practice. One father said, ‘I will point out the systematization in lining up specific strategies that are not too…, or that the strategies are simple to accomplish and not too extensive, and measurable. That has been useful, and something we had not done much of before’ (FC).

The parents’ perceived value of child participation differed. Two sets of parents having children with severe impairments felt that the intervention could have been performed without the child. Mother E said, ‘Then he doesn’t quite remember, and then he spends a very long-time processing and figuring out what to say. So, I think maybe he didn’t benefit as much’ (ME). The remaining parents highlighted child participation as a central success factor. One mother reflected, ‘The goal had to be important for the child; if not, it would have been harder to engage the child’. Another father said, ‘It was very important to us because … to include (the child) in the team… it is important to focus on something that is important to him’ (FA). All parents denied that the intervention had been too problem-focused but acknowledged that it was difficult for their child to be entirely positive about the intervention:

‘We as parents see a clear benefit and a structure in her rehabilitation that has been brilliant. She finds it boring, yes, she thinks so, but she would probably have found every other similar thing tiring anyway’. (FF)

Some parents considered improved cooperation with the child’s school as the most valuable part of the CICI. Some parents joined the school meetings. One father described his experience as follows: ‘…the school meetings that I have been in lately have been completely crucial for what’s offered. In that sense, we’ve been super lucky! It’s (…) been important’ (FF).

The parents pointed out the benefit of the combination of family and school sessions. Parents who earlier felt that the school did not understand their child’s challenges and needs were relieved: ‘We thought; okay, we’re not getting anywhere here (with the school). It just… must be like this. But then the CICI study came along with both Statped and their knowledge of all this. And now the teachers are really on board with us’ (MD).

The number of meetings was acceptable, apart from one father who felt there were too many since the seventh session had less content than the previous sessions. Most parents felt that the digital meetings worked well for them, but that the children would have benefitted more from physical meetings.

#### Teachers’ experiences of acceptability

Most teachers experienced that the CICI provided them with a greater understanding of ABI and awareness of the students’ challenges. However, they sometimes found it difficult to understand certain aspects of ABI. Teacher D said,

‘I have never seen fatigue in school before, so that is another diagnosis she maybe has acquired in a way. So, I had never heard about that before. That very small breaks can make such an enormous difference, I don’t know, that is a bit difficult for me to understand. It is a thing that you must choose to believe in, and not think logically about’.

This new insight helped them pay attention to the child’s challenges and need for adaptation at school. They felt that the intervention helped because all teachers involved in the child’s learning acquired a shared understanding. One teacher explained, ‘It has been a huge improvement for the student, although I do not think that she is fully aware of all the improvements we have facilitated for her during the school day’ (TB).

Nonetheless, a few teachers struggled to understand what the CICI comprised and questioned whether they helped treat the child’s brain injury and did not just facilitated education. One teacher explicitly stated, ‘Because it is the treatment of an injury we are doing in a way’ (TD).

The teachers perceived the intervention as valuable since it was provided by therapists with skills and competencies in the field. Teacher E: ‘Immediately, there was an experience of getting help from someone who had constructive suggestions right away’. Most teachers noted that they now had a shared understanding of the child’s challenges with the parents. The teachers also pointed out that the intervention led to an increased acknowledgement from parents that the teachers had already implemented adjustments prior to the study. Therefore, the teachers wanted the parents to attend the school meetings. One teacher said, ‘And then I think the parents could have been more involved in the school meetings, because then they could have spoken on behalf of the child’ (TC).

The teachers highlighted the timesaving aspect of being able to participate digitally. Although the teachers did not comment on the number of meetings, they thought that the intervention was time-consuming. Teacher E said, ‘Yes it took some time, but I have experienced these meetings as useful, right. I have been satisfied after every meeting, which is not something you normally say after a meeting. This was worthwhile. I am happy with the time I invested’. Finally, two teachers pointed to the dilemma of spending time helping one student at the cost of others, since the participating CICI student was not necessarily the one who struggled the most in the school.

Overall, the participants experienced the intervention as acceptable, useful, highly valuable and rewarding for most participants, particularly the parents.

## Discussion

The objective of this feasibility study was to improve the final randomized controlled trial (RCT) design to better understand how children, parents and teachers experienced participation and acceptability; to develop knowledge about the mechanisms of change, and to explore how the CICI was tailored to the context.

The study results indicated that the children, teachers and particularly parents felt involved and able to influence the decisions made in the intervention. They experienced the intervention as acceptable and found it mostly useful, valuable and rewarding.

Overall, the children who participated in the intervention experienced that their opinions were heard. All children felt acknowledged, which is an essential aspect of participation [25] and an assumed mechanism of change. The parents, teachers and therapists facilitated the child’s participation in a flexible way. However, involvement seemed challenging for children with severe cognitive impairment, exemplified by their need for extended support in the interviews and in the intervention sessions. Therefore, it is difficult to argue that their retrospective opinions fully represented their experiences of participation and acceptability at the time of participation. Participation can represent a particular challenge for this group due to the combination of attentional and other cognitive deficits, including a lack of awareness of their deficits [34]. Flexible attendance can be a realistic goal rather than expecting involvement from all children. However, children’s age and ability to attend and engage in the rehabilitation process must guide their participation in sessions. Sometimes, it can be a better choice to work indirectly through parents and/or teachers to structure the child’s environment or context to optimally support their functioning instead of working directly with the child. Nevertheless, in future RCTs, care should be taken to promote involvement, when possible, by, for example, giving frequent reminders of progress and by ensuring that the agreed-upon plans are acceptable and meaningful to the children. As opposed to attendance, involvement may include experiences of engagement, motivation, persistence, social connection and level of effect [12]. Although involvement in decisions about their goals increases patient motivation in their rehabilitation, patients vary in how much involvement they wish to have in their rehabilitation [35]. Furthermore, it is essential to facilitate attendance, since the probability of being involved in an activity increases if it is an activity that one frequently attends [36]. Nonetheless, a concern is that some children experienced that the adults did not act upon their contribution and that their parents´ opinions were weighted above theirs. One possible explanation may be that therapists took turns validating the children’s and the parents’ feelings, and furthermore, tried to establish common ground between the two. This may have left some children to experience that the adults were talking around them rather than with them. This can also be due to difficulties in adjusting and engaging the child in the mainly verbal intervention. Moreover, some children have limited awareness of their challenges, some have severe cognitive deficits, and some prefer not to talk about their injuries, resulting in a need to focus goals on adapting environmental factors through collaboration with parents, despite the children not being fully involved. The example with the earplugs demonstrates the following dilemma: when noise increases fatigue, motivating the child to try new or other more acceptable strategies to reduce fatigue can be meaningful, even though the child initially does not prefer them. However, children’s voices have intrinsic value [37], and continued attention by the CICI team to their impairments, level of insight and emotional maturity must be provided, balancing the child’s voice with the adults’ voice. Providing the children with a feeling that their opinions matter is probably a key factor in ensuring active participation from them.

The digital format affected the children’s experiences. As noted by parents and some of the children, the children could have experienced the meetings as more engaging if they were physical rather than the synchronous videoconference used. The videoconference format might have hindered alliance building, since eye contact is difficult, and the use of methods like games, drawings and physical objects is more difficult. In line with previous research [38, 39], the age and developmental stage of the children were important factors in determining children that best benefit from therapy through videoconferencing. In another study on digital rehabilitation, children who dropped out were more likely to have severe impairment following brain injury [38], indicating that such children are at higher risk of non-participation in digital rehabilitation interventions. In contrast, the digital format allowed children to participate safely from their own homes; in particular, it allowed children with severe fatigue to participate without the hassle of travelling. Tele-rehabilitation has demonstrated elevated levels of attendance by families in rehabilitation [40]. It is considered a strength of the intervention that the children are invited to participate in intervention sessions at the attendance or involvement level.

In most families, the children’s own participation and engagement in their rehabilitation was perceived as an important mechanism of change. However, children with ABI are often not included in family interventions [6]. For two of the children with the most severe impairment, their parents and teachers argued that the intervention would have provided the same outcome *without* the child’s participation or knowledge of the CICI. They perceived the strategies intended to adjust the child’s context as the most valuable. As such, particularly for children with severe impairments, the most prominent mechanism of change was probably not related to the direct involvement of the child in the sessions. Rather, for some families, the mechanisms of change can be found equally in the context (aim to establish compensatory strategies and environmental adaptations) and in the involvement of the child. This flexibility in involving the children to varying degrees can be considered a strength of the intervention, indicating how the CICI can also be promising for families with children whom suffer severe impairment, and therefore have limited ability to benefit from attending a rehabilitation meeting. In the RCT, continued efforts will be made to slow down and rephrase the exchanges to improve understanding and engagement. It is also important to closely regulate the time the child will be required to attend the digital meetings. The intervention was experienced as acceptable and especially useful and valuable to parents. It was judged acceptable by families regardless of the time since the child’s injury. The parents perceived that the intervention had helped the family in their everyday lives. These considerations of usefulness are in line with the recommendations from a recent review on including family-based intervention in the rehabilitation of children with ABI [6]. As mentioned, the participants highlighted different approaches that were most helpful, such as new knowledge and a SMART-goal approach, whereas many voted for the ‘whole package’. This indicates that the components described in Table 1 were experienced as useful and acceptable but also that they worked well together and hopefully reinforced each other in creating a change [41]. However, additional embedded mechanisms may contribute to a change in outcomes. For the participants to delineate explicitly which components of a complex intervention they find helpful is not expected, since this is a complex intervention designed to work on multiple levels and target a heterogeneous group of participants. When the participants said the ‘whole package’ of the CICI was the most helpful, it exemplifies how difficult it can be to delineate the active ingredients in rehabilitation since treatment often involves simultaneous application of multiple different treatments [42]. The level of individualization of the intervention may also influence their experience. Since the CICI is flexible, it can support the structural, personal and social processes of motivation and ability. In turn, this might reinforce behavioral engagement and problem solving, which can be useful in diverse ways for different families. Various aspects of the intervention might have been of varied importance for both the separate groups of participants and the individual children, parents, and teachers. For example, the mechanisms of change may have been CICI-specific components, such as the SMART-goal approach or increased collaboration with the school system. For others, it might have been the child’s participation. The most relevant component might also have varied according to what was noted as the most challenging ABI-related problem.

Some children and parents felt that the school neither understood the child’s challenges nor adjusted for them prior to the intervention, although the need for long-term school support for children with ABI is well known [43, 44]. The teachers perceived the intervention as useful and important for the children, as they received relevant support, learned about the students’ challenges and experienced a high degree of participation. Involving the schools in the intervention meant widening the possibilities for environmental adaptations, which was an important aspect of the intervention. The involvement of schools was also central to parents’ acceptability of the intervention. Few educators have the training and knowledge needed to adequately monitor and ensure the fit between the students’ learning needs and environmental adaptations [45], despite schools regularly serving as long-term service providers [46]. At the same time, the teachers’ class perspective made them elaborate on the time spent on this student as opposed to other students with just as legitimate needs. Moreover, the value of the CICI was more unclear to the schools than to the parents. The teachers acknowledged that the intervention benefitted the children and their well-being, but that it was difficult to fully understand the children’s need for academic support. The logic of treatment and rehabilitation can be difficult to translate into the logic of education [47]. In future RCTs, continued attention to the challenges of integrating perspectives from the health and educational systems should be prioritized. However, some of the teachers’ challenges to acceptability were related to a lack of understanding from their school leaders, which is related to the school organization and not the intervention per se. There is a need to increase school buy-in and engagement and better educate school leadership, so they are willing to free up teacher time to participate. In the final RCT, efforts will be made to invite, better inform, and retrieve a clear consent for participation from the teachers’ leaders.

### Methodological considerations

One strength of the interview study was the inclusion of children, parents, and teachers. For some children, their impairment made it difficult to provide answers to the questions during the interviews but was enabled by their parents, as described previously. Therefore, it is sometimes difficult to delineate to what degree the parents’ experiences affected the answers for three of the children.

A strength of the feasibility study was that the children participated to a large degree, although from the perspectives of the parents and teachers of the most severely injured children, the intervention’s usefulness did not necessarily seem to depend on child participation. The feasibility study included neither families of children whose parents did not live together or single-parent households nor how the intervention might be working if the schools chose not to participate. In addition, the participating children in this study skewed older than the full age range for the intervention, the experiences of younger children is unknown. The consequences this will have on the experience of participation, acceptability and what mechanisms of change might be working must be addressed in future evaluations of the full RCT. Future qualitative studies targeting these groups may be warranted prior to implementing the full RCT. To offer an appropriate rehabilitation path to the individual child, future rehabilitation research should continue to investigate the combination of minimum cognitive abilities, level of awareness and emotional readiness for them to be able to engage successfully in a particular treatment such as the CICI.

## Concluding remarks

The intervention was perceived as acceptable, with a high degree of experienced participation for all participants, especially the parents. It was feasible for families of children with diverse levels of impairment and time since injury. The intervention components were experienced as useful, valuable, and of different importance depending on the families’ self-reported main ABI-related problems in daily life. Hence, the intervention is flexible and allows for an individualized approach. The intervention facilitated different mechanisms of change and can be tailored to different contexts. The digital format of the intervention allowed for participation regardless of geographical distances, saved the therapists and participants’ time and allowed for flexibility regarding the amount of participation from the children.

## Data Availability

The datasets generated and analyzed during the current study are not publicly available due to the risk of compromising individual privacy.

## Abbreviations

ABI: Acquired Brain Injury
CICI: Child in Context Intervention
CT: Computed Tomography
MRI: Magnetic Resonance Imaging
REK: Regional Ethics Committee
RCT: Randomised Controlled Trials
TBI: Traumatic Brain Injury
